# Supplementary material for: An adeno-associated virus variant enabling efficient ocular-directed gene delivery across species
Source: Nat Commun. 2024 May 6;15:3780. doi: 10.1038/s41467-024-48221-4 (PMC11074261; doi:10.1038/s41467-024-48221-4)
Supplement: Supplementary file 1 — Supplementary information [file 41467_2024_48221_MOESM1_ESM.pdf]

Supplementary information

Supplementary Table.1 | AAV8 variants information

Name	Mutation	Name	Mutation
v120	Q588~A592→RGNRQ	v129	Q588~A592→RGNQQPRPTSRQ
v121	Q588~A592→QQNTARGNRQ	v1210	Q588~A592→SGNQQPRPTSTQ
v122	Q588~A592→RGNRQAAQQNTA	v1211	Q588~A592→RGNQQPTPSTRQ
v123	Q588~A592→RGNRQQNTA	v1212	Q588~A592→SGNQQPTPSTTQ
v124	Q588~A592→RGNRQQQNTA	v1213	Q588~A592→RGNRQAAQQPTPTS
v125	Q588~A592→SGNTQ	v1214	Q588~A592→RGNRQQPTPTS
v126	Q588~A592→SGNTQAAQQNTA	v1215	Q588~A592→QQNTAQQPTPTS
v127	Q588~A592→SGNQQNTATQ	v13	N263~T274→SQSGASNDNH
v128	Q588~A592→RGNQQNTARQ	v1312	N263~T274→SGTGASTDTH

Supplementary Table.2 | Primer pairs for AAV8 mutagenesis

Mutation in AAV8	Forward primer (5'-3') (lowercase=mutation bases)	Reverse primer (5'-3')
v120	aacagacaaCCTCAAATTGGAACTGTCAAC	gcctctCTGCAAGTTATCTGCCACGATACCG
v121	acagacaacCTCAAATTGGAACTGTCAACAGCC	tgcctcTAGCCGTGTTTTGCTGCTGCAAG
v122	acaagccgctCAGCAAAACACGGCTCCTCAAATTG	ctgttgcctctCTGCAAGTTATCTGCCACGATACC
v123	aacagaCAACAAAACACGGCTCCTCAAATTG	gcctctCTGCAAGTTATCTGCCACGATACC
v124	acagacaaCAGCAAAACACGGCTCCTCAAATTG	tgcctctCTGCAAGTTATCTGCCACGATACC
v125	cactcaaCCTCAAATTGGAACTGTCAACAGC	ttgccgctCTGCAAGTTATCTGCCACGATAC
v126	tcaagccgctCAGCAAAACACGGCTCCTCAAATTG	gtgttgccgctCTGCAAGTTATCTGCCACGATACC
v127	aacacggctactcaaCCTCAAATTGGAACTGTCAACAGC	ttgctggttgccgctCTGCAAGTTATCTGCCACGATACCGT
v128	aacacggctagacaaCCTCAAATTGGAACTGTC	ttgctggttgcctctCTGCAAGTTATCTGCCACG
v129	agaccaaccagcagacaaCCTCAAATTGGAACTGTCAACAGCC	aggttgctggttgcctctCTGCAAGTTATCTGCCACGATACCGTG
v1210	agaccaaccagcactcaaCCTCAAATTGGAACTGTCAACAGCC	aggttgctggttgccgctCTGCAAGTTATCTGCCACGATACCGTG
v1211	accccaagcaccagacaaCCTCAAATTGGAACTGTCAACAGCCA	aggttgctggttgcctctCTGCAAGTTATCTGCCACGATACCG
v1212	accccaagcaccacccaaCCTCAAATTGGAACTGTCAACAGCC	aggttgctggttgccgctCTGCAAGTTATCTGCCACGATACCG
v1213	cagcaacctaccccaaccagcCCTCAAATTGGAACTGTCAACAGCC	agctgcctgtctgttgcctctCTGCAAGTTATCTGCCACGATACCG
v1214	caacctaccccaaccagcCCTCAAATTGGAACTGTCAACAGCC	ctgctgtctgttgcctctCTGCAAGTTATCTGCCACGATACCG
v1215	cccaaccagcCCTCAAATTGGAACTGTCAACAGCCAGG	gtaggttgctgagccgtgttttgctgCTGCAAGTTATC
v13	tcgaacgacaatcacTACTTCGGCTACAGCACCCCC	ggctcctgattggctGGAGATTTGCTTGTAGAGGTG
v1312	tcgaccgacacccacTACTTCGGCTACAGCACCCCC	ggctccggtgccgctGGAGATTTGCTTGTAGAGGTG

Supplementary Table.3 | The mRNA expression levels of AAV8 and AAV8 variants after intravitreal injection

Mutation in AAV8	mRNA expression level
AAV8	1.0±0.1
v120	1.8±0.62
v121	33.57±2.26
v122	3.33±1.25
v123	13.57±2.15
v124	8.53±2.06
v125	19.97±3.31
v126	0.83±0.35
v127	0.57±0.21
v128	75.37±5.06
v129	5.57±1.7
v1210	2.87±0.75
v1211	0.87±0.4
v1212	1.73±0.71
v1213	9.47±1.96
v1214	0.8±0.46
v1215	2.07±1.06
v13	2.63±1.16
v1312	0.87±0.35

Supplementary Table.4 | Concentrations of the transgene product (anti-VEGF protein) in different tissues of rhesus monkeys after a single suprachoroidal injection in bilateral eyes (ng/g for solid tissues or ng/mL for liquid tissues)

No.	Aqueous humor*		Choroid		Conjunctiva		Iris/Ciliary body		Retina		Sclera		Vitreous body*	
	OS	OD	OS	OD	OS	OD	OS	OD	OS	OD	OS	OD	OS	OD
AAV8	BLQ	BLQ	31.38	BLQ	BLQ	BLQ	41.22	55.32	37.68	50.82	BLQ	BLQ	BLQ	BLQ
AAVv128	9.95	12.1	1032	1638	BLQ	48.48	502.2	134.4	2112	4056	84	48.42	36	44.9

Note: "*" means liquid tissues, with the unit of ng/mL; "–" means no tissue; BLQ means below the lower limit of quantification.

Supplementary Table.5 | Concentrations of the transgene product (anti-VEGF protein) in aqueous humor of rhesus monkeys after a single suprachoroidal injection in bilateral eyes (ng/mL)

No.	1F001		1F002		1M001		1M002		4F001		4F002		4M001		4M002	
	AAV8								AAVv128							
	OS	OD	OS	OD	OS	OD	OS	OD	OS	OD	OS	OD	OS	OD	OS	OD
	BLQ	BLQ	BLQ	BLQ	BLQ	BLQ	BLQ	BLQ	88.2	39.3	172.1	189.7	119.5	57.8	24.6	26.9

Note: BLQ means below the lower limit of quantification.

Supplementary Table.6｜Grade IV CNV lesions % of each assessable eye at day 35 and day 49 (dose: 2×10^{12} vg/eye, 100 μL volume, n=8)

Time	Vehicle			AAV8			AAVv128		
	No.	spots	Grade IV lesions (%)	No.	spots	Grade IV lesions (%)	No.	spots	Grade IV lesions (%)
Day35	5F001-OD	2	33.33	1F001-OD	6	100.00	4F001-OD	0	0.00
	5F001-OS	3	50.00	1F001-OS	3	50.00	4F001-OS*	N/A	N/A
	5F002-OD	5	83.33	1F002-OD	1	16.67	4F002-OD	0	0.00
	5F002-OS	5	83.33	1F002-OS	3	50.00	4F002-OS	0	0.00
	5M001-OD*	3	60.00	1M001-OD	0	0.00	4M001-OD	0	0.00
	5M001-OS	1	16.67	1M001-OS	0	0.00	4M001-OS	0	0.00
	5M002-OD	2	33.33	1M002-OD	0	0.00	4M002-OD	0	0.00
	5M002-OS*	0	0.00	1M002-OS	2	33.33	4M002-OS	0	0.00
Day49	5F001-OD	6	100.00	1F001-OD	5	83.33	4F001-OD	0	0.00
	5F001-OS	4	66.67	1F001-OS	4	66.67	4F001-OS*	N/A	N/A
	5F002-OD	6	100.00	1F002-OD	2	33.33	4F002-OD	0	0.00
	5F002-OS	5	83.33	1F002-OS	3	50.00	4F002-OS	0	0.00
	5M001-OD	5	83.33	1M001-OD	0	0.00	4M001-OD	0	0.00
	5M001-OS	5	83.33	1M001-OS	1	16.67	4M001-OS	0	0.00
	5M002-OD	3	50.00	1M002-OD	1	16.67	4M002-OD	0	0.00
	5M002-OS	0	0.00	1M002-OS	4	66.67	4M002-OS	0	0.00

Note: Six laser spots were applied for each eye. Grade IV CNV lesions % of each assessable eye was calculated as follow: Grade IV CNV lesions % = the absolute number of grade IV lesions ÷ the total number of assessable lesions. N/A: The laser spot could not be observed due to bleeding after laser modelling. It was not included in the statistical values.

Supplementary Table.7｜ Statistics for Cryo-EM data collection, image processing and model building

	AAVv128
**PDB entry**	8JRE
**EMDB entry**	EMD-36594
**Data collection and processing**	
Images (no.)	2,041
Magnification	165,000
Voltage (keV)	300
Electron exposure (e^{-}/Å^{2})	50
Defocus range (μm)	-1.2 to -2.4
Pixel size (Å)	0.73
Symmetry imposed	I
Final particle images (no.)	90,744
Map global resolution (Å)	2.08
Resolution FSC threshold	0.143
**Refinement**	
Model resolution (Å)	2.20
FSC threshold	0.5
Map sharpening *B* factor (Å^{2})	-20
R.m.s. deviations	
Bond lengths (Å)	0.006
Bond angles (°)	1.024
Validation	
MolProbity score	1.23
Clashscore	4.56
Ramachandran plot	
Outliers	0.00
Allowed	1.72
Favored	98.28
Model vs. Data	
CC (mask)	0.92
CC (volume)	0.90

Supplementary Table.8｜ The hydrogen bonding interactions within VR-III loop.

AAVv128	
L586	R596
R587	R596
R588	T594, R596
G589	N593, A595
N590	A595
AAV8	
Q587	T591
Q588	T591

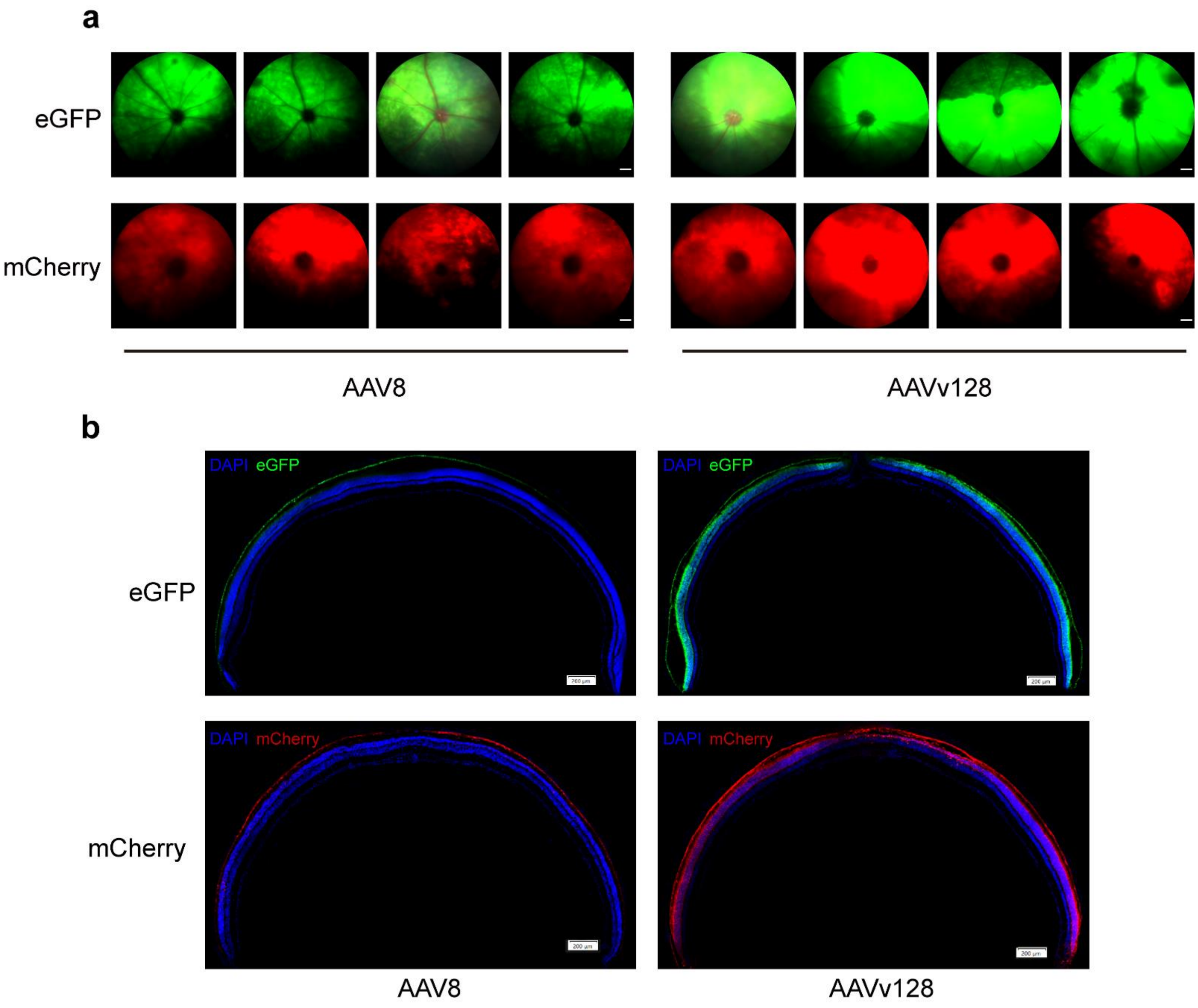

Supplementary Fig. 1 | Evaluating the transduction of the AAV8 and AAVv128 capsids with different transgenes after subretinal injection.

a, Fluorescence fundoscopy of mouse eyes treated with ssAAV-CBA-eGFP/mCherry vectors packaged with AAV8 or AAVv128 capsids (1×10^{9} vg/eye, 1 μL volume, n=4). Mice were imaged at D28 post-injection, and mice were sacrificed at D28 and eyes were harvested. Scale bar: 500 μm. **b**, Coronal sections of mice retina transduced with rAAV-CBA-eGFP/mCherry. Native eGFP expression (green) or mCherry expression (Red) shows the positively transduced cell. Scale bar: 200 μm

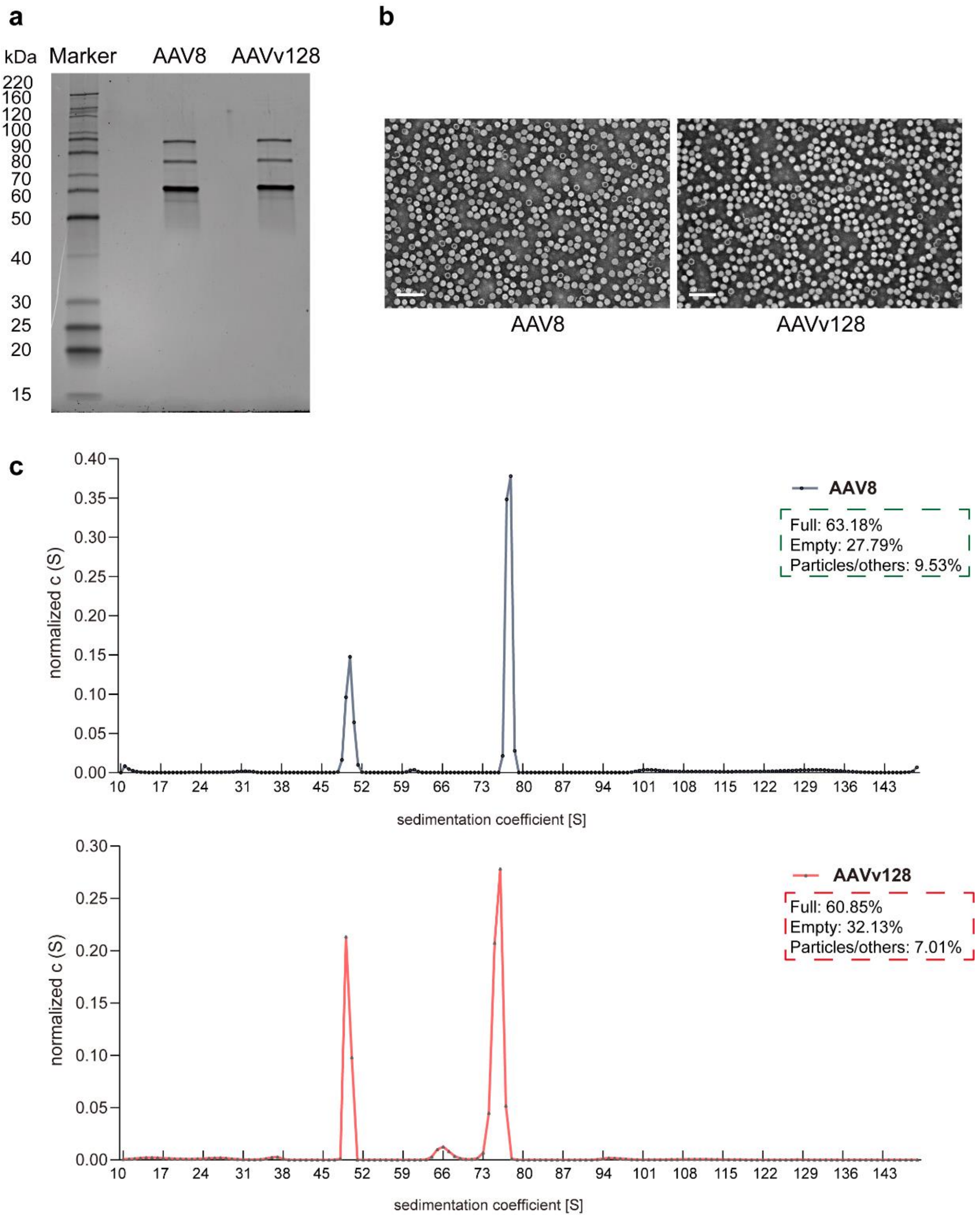

Supplementary Fig.2 | The quality of AAV8 and AAVv128 samples for evaluating the treatment efficacy for nAMD studies. **a**, SYPRO Ruby-stained SDS-PAGE analysis of anion exchange chromatography-purified AAV8 and AAVv128. **b**, Transmission electron microscopy (TEM) micrographs of AAV8 and AAVv128. Full (bright spheres) and empty (spheres with darker spot). **c**, rAAV samples were analyzed by using Sedimentation velocity analytical Ultracentrifugation (SV-AUC) to determine the AAV8 or AAVv128 empty-to-full capsid ratio. Scale bar: 100 nm

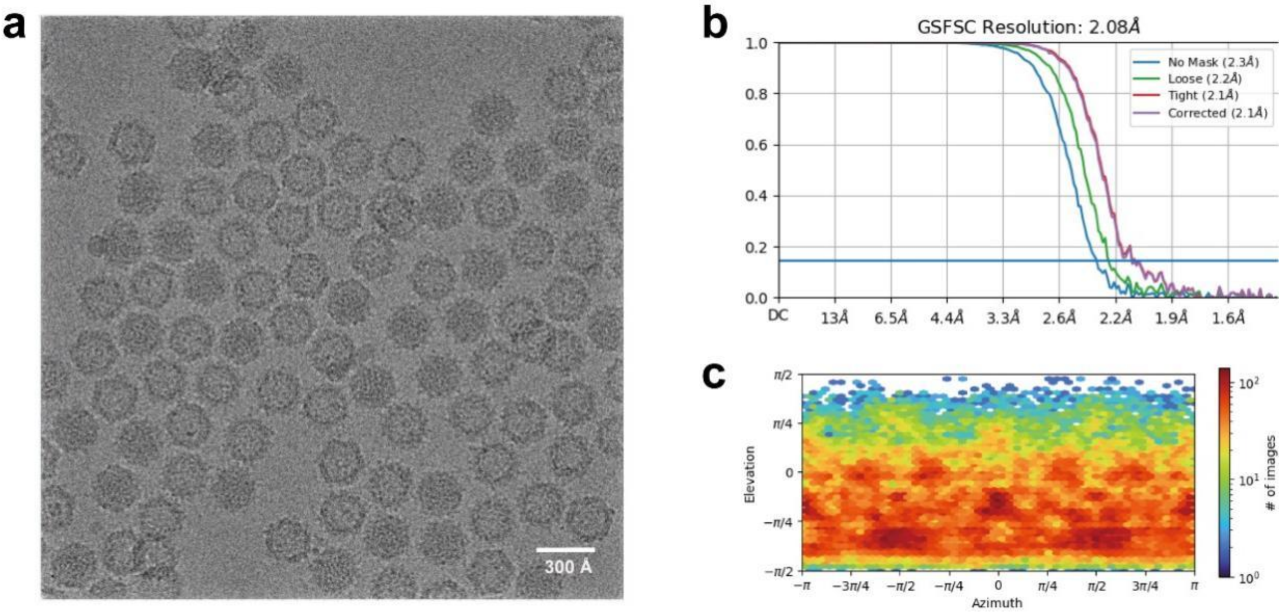

Supplementary Fig.3 | Cryo-EM structure determination of AAVv128.

a, Representative micrograph image of AAVv128. Scale bars: 300 Å. **b**, Gold Standard Fourier Shell Correlation (GSFSC) curve. **c**, Direction distribution map of particles.

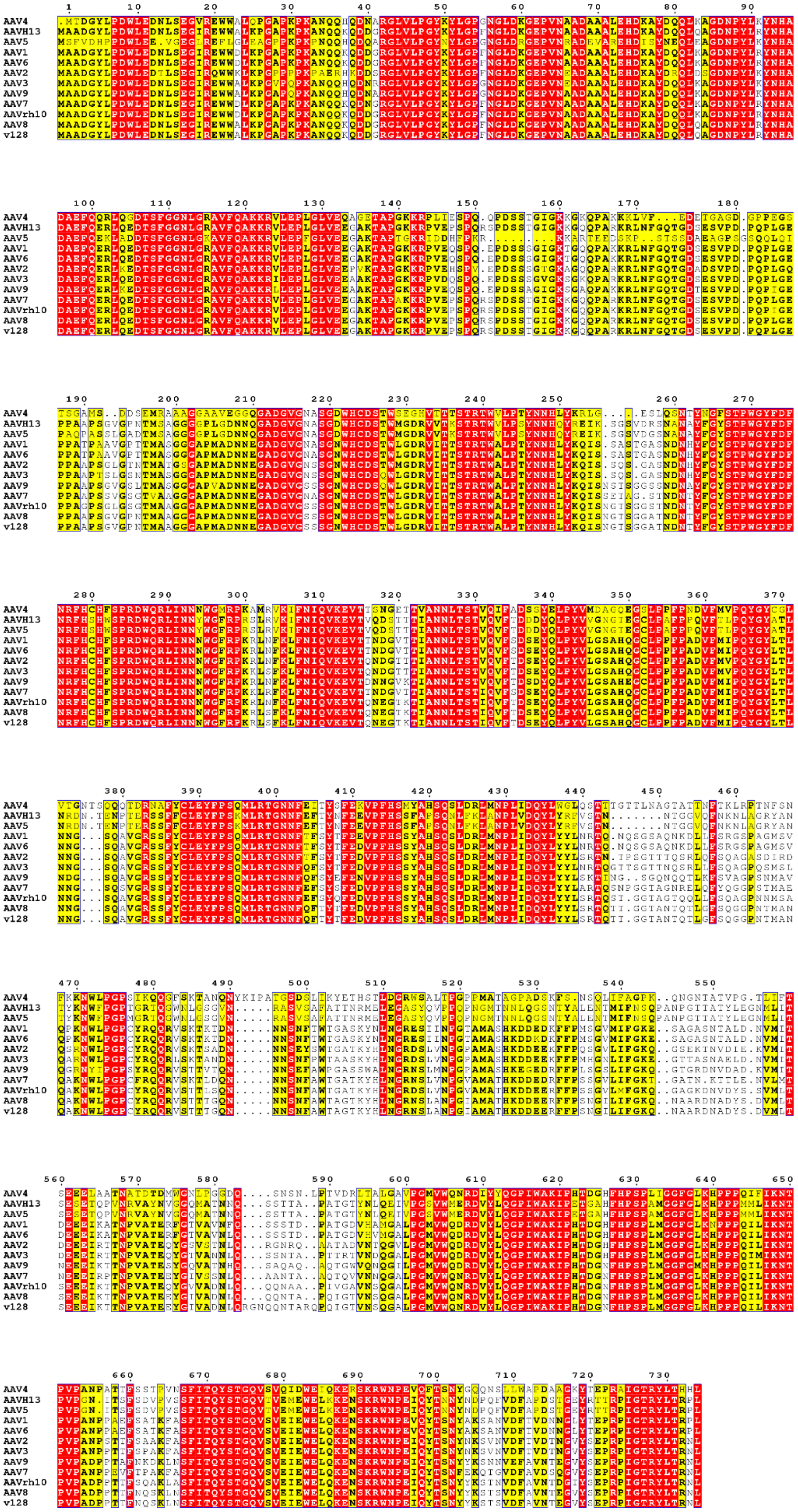

Supplementary Fig.4 | Amino acid sequence of the AAVs/AAVv128 capsid.

The identical or conserved residues are shown as letters with red or yellow backgrounds, while the non-conserved residues have a white background.

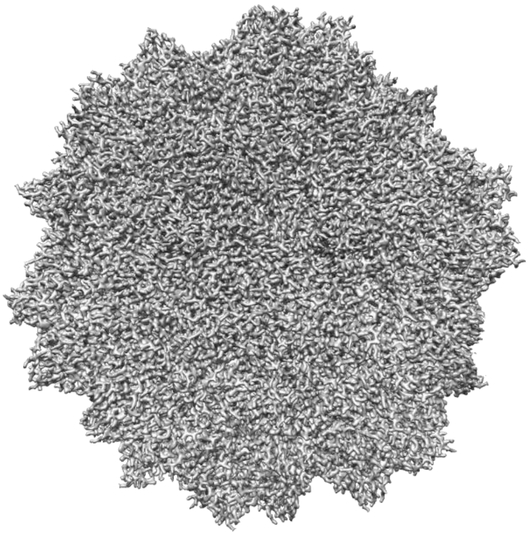

Supplementary Fig.5 | Electron density map of AAVv128 capsid.

The electron density map was produced in Chimera and was shown in grey.

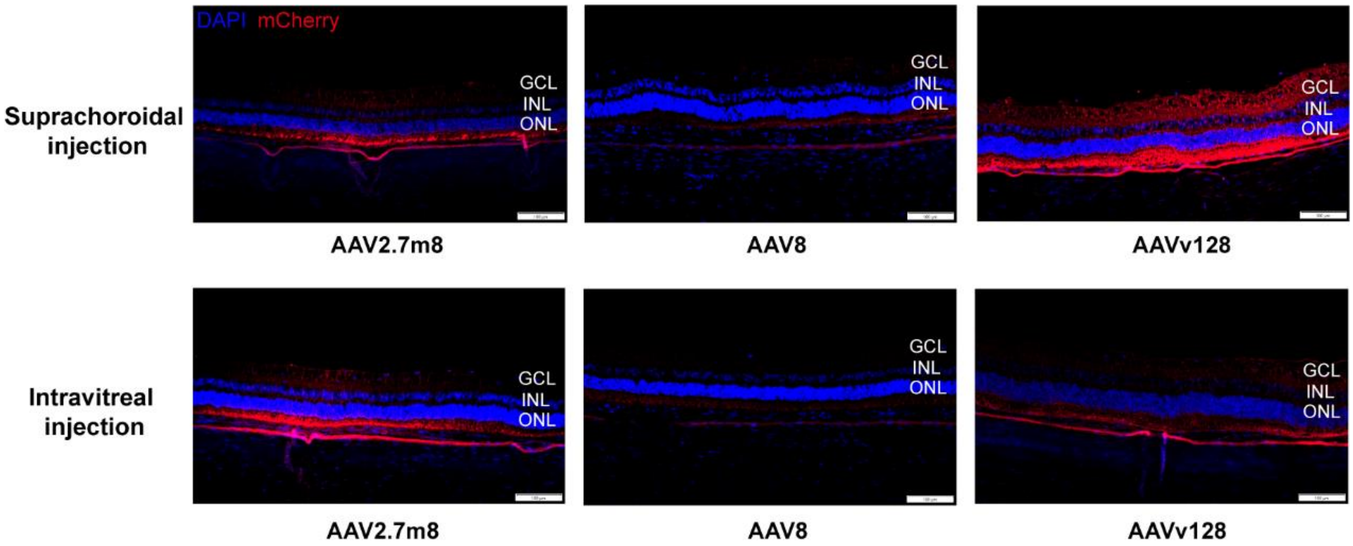

Supplementary Fig.6 | Intraocular injections in New Zealand rabbits to evaluate the transduction efficacy of AAV8, AAVv128 and AAV2.7m8

The New Zealand rabbit eyes treated by intravitreal injections or suprachoroidal injections of the AAV8 and AAVv128 confers detectable mCherry expression at days 28 post-injection (1×10^{11} vg/eye, 100 μL volume). Scale bars: 100 μm

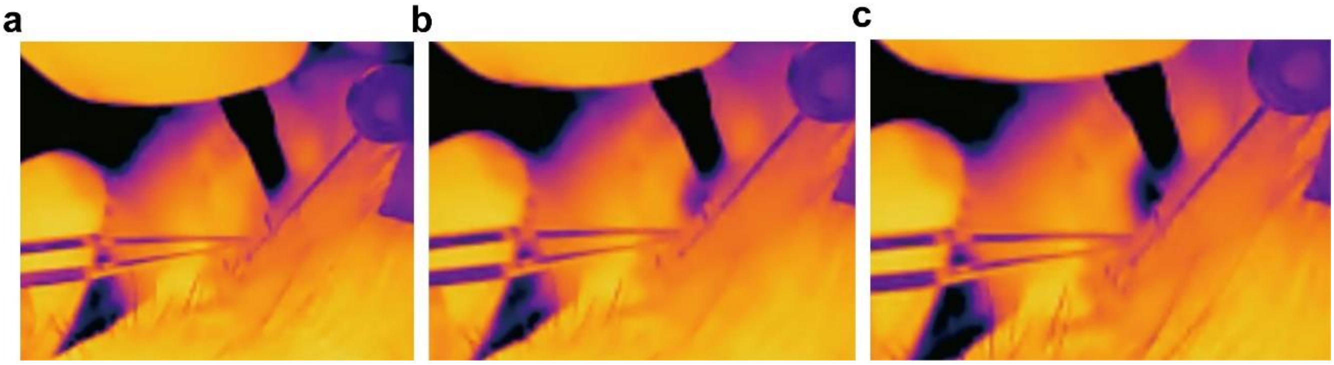

Supplementary Fig.7 | Infrared imaging analysis of suprachoroidal injections in cynomolgus monkeys.

The SCS injection process was monitored by using an infrared thermal camera. The early (**a**), middle (**b**), and late (**c**) stages of the injection process were shown.

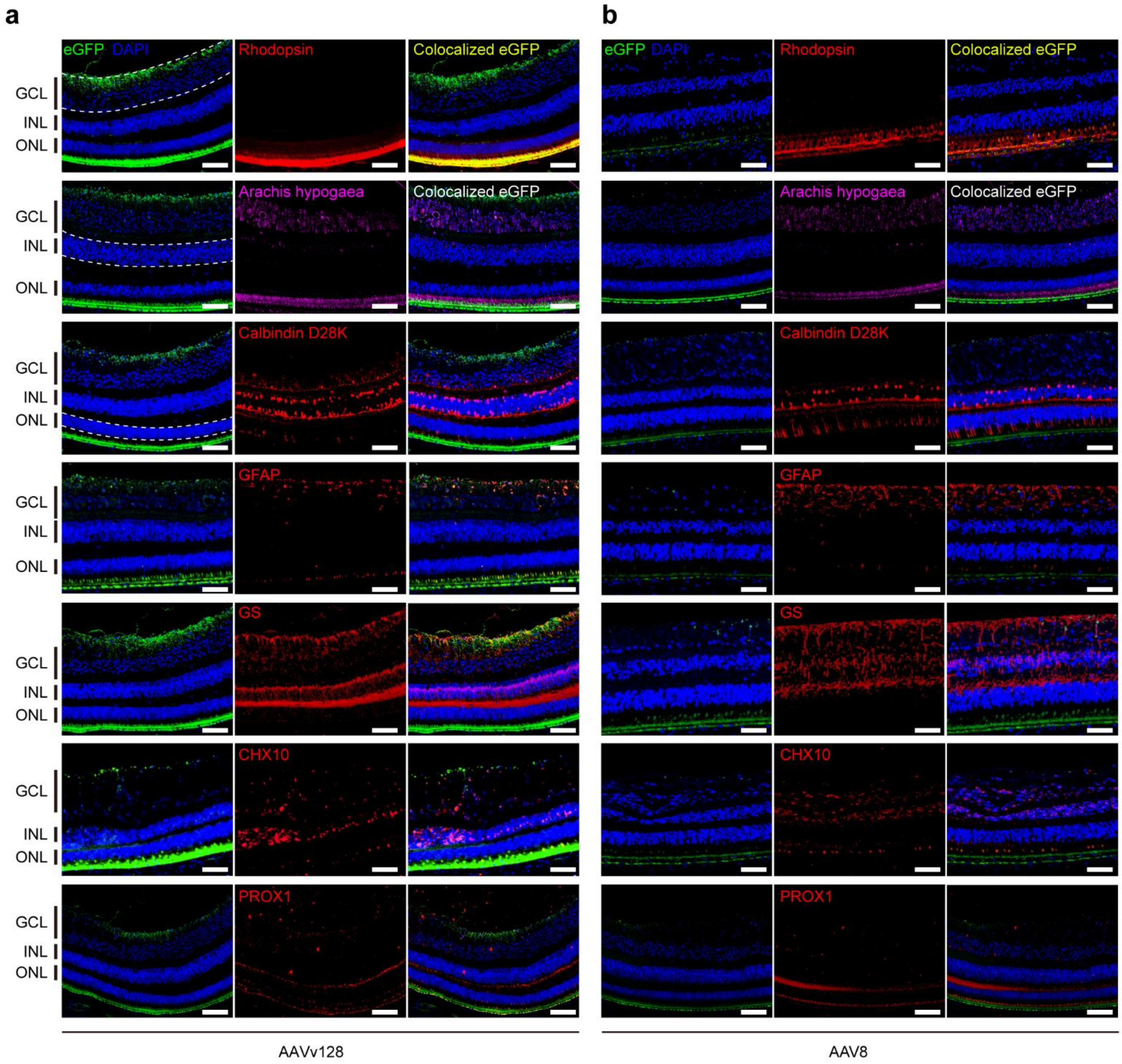

Supplementary Fig.8 | Cross-section and immunofluorescence analyses of NHPs retina treated with suprachoroidal injection of AAVv128 and AAV8.

NHPs eyes treated by suprachoroidal injection of the AAVv128 (**a**) and AAV8 (**b**) capsid (3.5×10^{12} vg/eye, 100 μL volume, n=1). NHPs where sacrificed at D14 and eyes were harvested. Coronal sections of rAAV-CB-eGFP transduced NHPs retina. Immunofluorescence stained sections (red or purple) with antibodies against Rhodopsin (photoreceptor cells, Rod), Arachis hypogaea (optic vertebral cells), Calbindin D28K (horizontal cells, anaplastic cells and ganglion cells), GFAP (astrocytes), GS (Müller cells), CHX10 (bipolar cells), or PROX1 (anaplastic cells and ganglion cells) indicate the distribution of cell types across the retina. Native eGFP expression (green) that colocalize with IF staining (yellow or white) reveals the positively transduced cell type indicated. Scale bars: 50 μm; GCL: ganglion cell layer, INL: inner nuclear layer, ONL: outer nuclear layer.

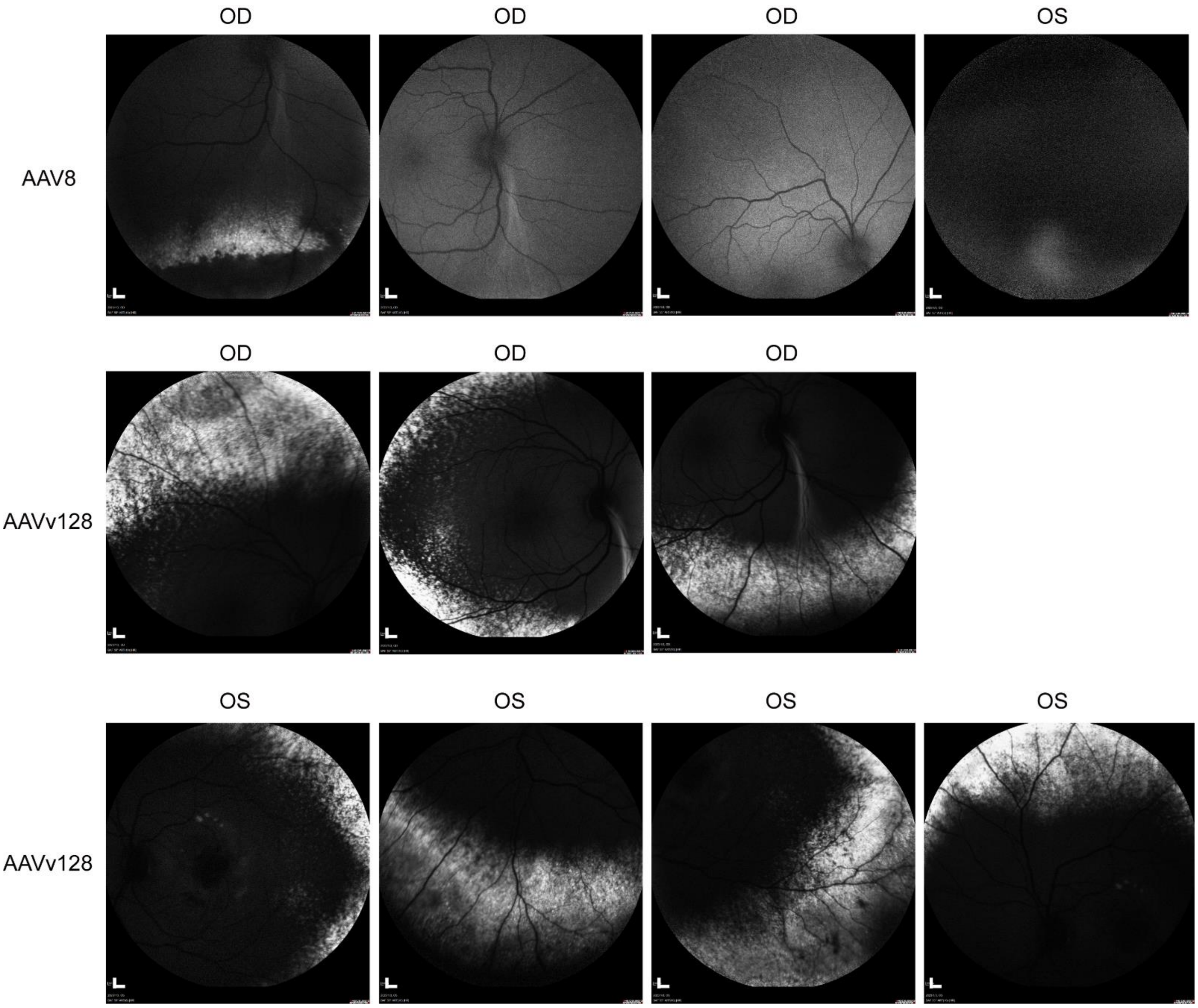

Supplementary Fig.9 | Transduction validation of NHPs eyes treated with AAV8 and AAVv128 by Scanning laser ophthalmoscopy (SLO).

NHPs eyes treated by suprachoroidal injection of the AAV8 and AAVv128 capsid confers detectable eGFP expression at days 14 post-injection (3.5×10^{12} vg/eye, 100 μL volume, n=1). OD, oculus dextrus; OS, oculus sinister; scale bars: 200 μm.

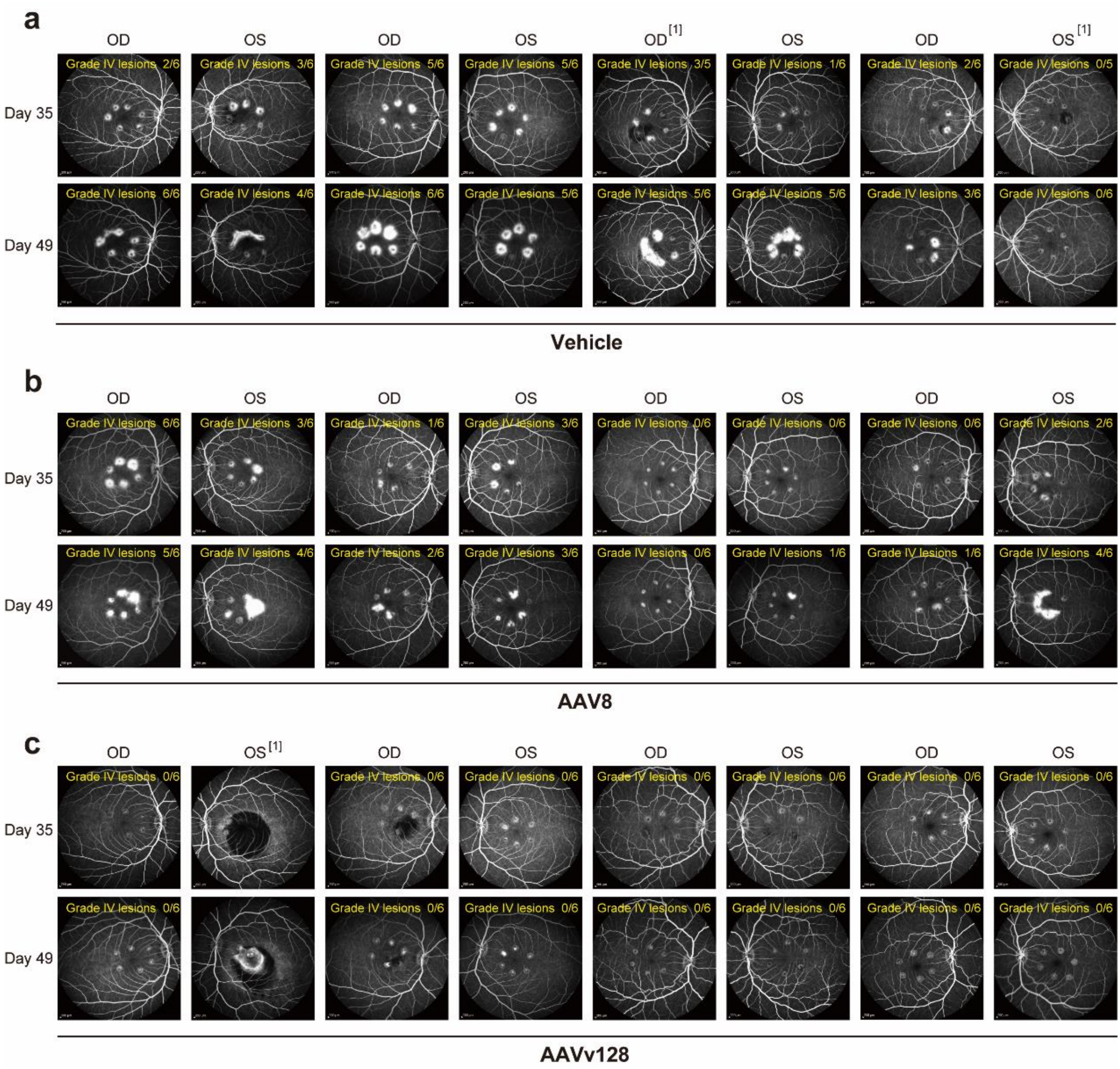

Supplementary Fig.10 | A model of laser-induced CNV in NHPs is being used to evaluate the efficacy of nAMD. Fluorescein fundus angiograph (FFA) was used to determine the number of Grade IV lesions. Representative FFA of NHP eyes treated with suprachoroidal injection of Vehicle (**a**), AAV8-anti-VEGF vector (**b**)and AAVv128-anti VEGF vector (**c**) at 35-days and 49-days (2×10^{12} vg/eye, 100 μL volume, n=8). OD, oculus dextrus; OS, oculus sinister; FFA, fundus fluorescence angiography. [1] The laser spot could not be observed due to bleeding after laser modelling. It was not included in the statistical values.

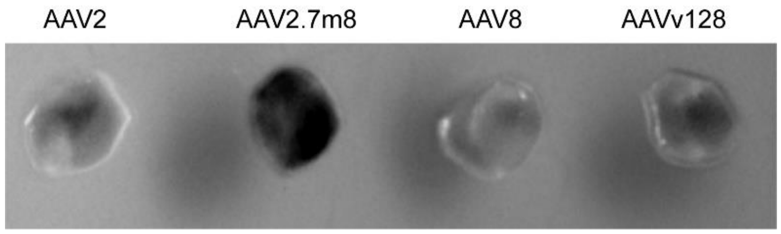

Supplementary Fig.11 | A heparin binding assay for AAV2, AAV2.7m8, AAV8 and AAVv128.
Heparin binding profile of AAV2, AAV2.7m8, AAV8 and AAVv128 (rAAV: 200 μL,1×10^{10} vg/mL).
