# Supplementary material for: An adeno-associated virus variant enabling efficient ocular-directed gene delivery across species
Source: Nat Commun. 2024 May 6;15:3780. doi: 10.1038/s41467-024-48221-4 (PMC11074261; doi:10.1038/s41467-024-48221-4)
Supplement: Supplementary file 3 — Description of Additional Supplementary Files [file 41467_2024_48221_MOESM3_ESM.pdf]

Description of Additional Supplementary Files

Supplementary Movie.1: Observation NHP suprachoroidal injection using the infrared camera
