# Supplementary material for: An adeno-associated virus variant enabling efficient ocular-directed gene delivery across species
Source: Nat Commun. 2024 May 6;15:3780. doi: 10.1038/s41467-024-48221-4 (PMC11074261; doi:10.1038/s41467-024-48221-4)
Supplement: Supplementary file 5 — Reporting Summary [file 41467_2024_48221_MOESM5_ESM.pdf]

nature portfolio

Corresponding author(s): Xun Sun
Qiang Zheng
Xiao Ke

Last updated by author(s): Mar 28, 2024

Reporting Summary

Nature Portfolio wishes to improve the reproducibility of the work that we publish. This form provides structure for consistency and transparency in reporting. For further information on Nature Portfolio policies, see our Editorial Policies and the Editorial Policy Checklist.

Statistics

For all statistical analyses, confirm that the following items are present in the figure legend, table legend, main text, or Methods section.

n/a	Confirmed	
☐	☒	The exact sample size (*n*) for each experimental group/condition, given as a discrete number and unit of measurement
☐	☒	A statement on whether measurements were taken from distinct samples or whether the same sample was measured repeatedly
☐	☒	The statistical test(s) used AND whether they are one- or two-sided<br>*Only common tests should be described solely by name; describe more complex techniques in the Methods section.*
☐	☒	A description of all covariates tested
☐	☒	A description of any assumptions or corrections, such as tests of normality and adjustment for multiple comparisons
☐	☒	A full description of the statistical parameters including central tendency (e.g. means) or other basic estimates (e.g. regression coefficient) AND variation (e.g. standard deviation) or associated estimates of uncertainty (e.g. confidence intervals)
☐	☒	For null hypothesis testing, the test statistic (e.g. *F*, *t*, *r*) with confidence intervals, effect sizes, degrees of freedom and *P* value noted<br>*Give P values as exact values whenever suitable.*
☒	☐	For Bayesian analysis, information on the choice of priors and Markov chain Monte Carlo settings
☒	☐	For hierarchical and complex designs, identification of the appropriate level for tests and full reporting of outcomes
☒	☐	Estimates of effect sizes (e.g. Cohen's *d*, Pearson's *r*), indicating how they were calculated

Our web collection on statistics for biologists contains articles on many of the points above.

Software and code

Policy information about availability of computer code

Data collection

1) Cryo-EM data was collected using H2/O2 glow-discharged 300-mesh Quantifoil R 1.2/1.3 grids (Quantifoil, Micro Tools GmbH, Germany), and subsequently blotted using a FEI Vitrobot and then frozen in liquid ethane. The grids were imaged on a Thermo Fisher Krios G4 microscope (Thermo Fisher Scientific, USA) equipped with a cold field-emission gun, a Selectris X energy filter and a Falcon 4 detector. The energy filter was operated with a slit width of 10 e-V to remove inelastically scattered electrons. Image stacks were collected using EPU software at a pixel size of 0.73 Å/pixel with a total dose of 50 e-/Å2.
2) Retinal section images were acquired on Olympus SpinSR10 spinning disc confocal super-resolution microscope (JAPAN, OlyVIA). Global retinal images (10X) and high-magnification images (60X region specific areas) were collected at the same intensity and exposure thresholds for each respective magnification.
3) OCT imaging was performed using the Micron ? retinal imaging system (phoenix micron IV).
4) Non-human primates (NHPs) underwent SLO using the Spectralis HRA OCT (Heidelberg). Confocal SLO was used to capture fluorescence images using 488-nm excitation light. Images were captured from the central macula and from the peripheral retina in regions of visible eGFP fluorescence by manually steering the Spectralis HRA OCT. For FFA examination, imgae acquisition was performed by Spectralis HRA (Heidelberg). Both early-phase (within approximately 1 min) and late-phase (after approximately 5 min) fundus angiograms were acquired.

Data analysis

1) Particles were picked automatically and were extracted with a box size of 448 pixels. After several rounds of 2D and 3D classifications, particles were used to perform Ab-initio Reconstruction and Heterogenous Refinement. The candidate model and particles were selected and refined using Homogeneous Refinement with I symmetry applied to generate the final cryo-EM map at a 2.08 Å resolution. Local resolution ranges were also analyzed within cryoSPARC according to the gold-standard Fourier shell correlation (FSC) cut-off of 0.143.
2) The atomic model was built based on the structure of AAV8 (PDB: 6V12). The structure of AAV8 was docked into the EM density map by using UCSF Chimera, this was then followed by iterative manual fitting adjustment in Coot and real space refinement in PHENIX. The VR-VIII loop was manually mutated to the corresponding amino acids in the model. All figures were made using UCSF ChimeraX and PyMOL.

3) The fluorescence pixel intensity and mean pixel intensity per pixel area were quantified using Image J (National Institutes of Health, NIH).
4) Graphs and statistical calculations were conducted in Prism 8 (GraphPad Software, Inc., San Diego, CA).

For manuscripts utilizing custom algorithms or software that are central to the research but not yet described in published literature, software must be made available to editors and reviewers. We strongly encourage code deposition in a community repository (e.g. GitHub). See the Nature Portfolio guidelines for submitting code & software for further information.

Data

Policy information about availability of data

All manuscripts must include a data availability statement. This statement should provide the following information, where applicable:

- Accession codes, unique identifiers, or web links for publicly available datasets
- A description of any restrictions on data availability
- For clinical datasets or third party data, please ensure that the statement adheres to our policy

Capsids and vector constructs described in this report will be made available upon request. The model and map of AAVv128 will be available from the RCSB Protein Data Bank PDB: 8JRE (https://doi.org/10.2210/pdb8jre/pdb) and the Electron Microscopy Database: EMD-36594 (https://www.ebi.ac.uk/emdb/EMD-36594), respectively.

Research involving human participants, their data, or biological material

Policy information about studies with human participants or human data. See also policy information about sex, gender (identity/presentation), and sexual orientation and race, ethnicity and racism.

Reporting on sex and gender	N/A
Reporting on race, ethnicity, or other socially relevant groupings	N/A
Population characteristics	N/A
Recruitment	N/A
Ethics oversight	N/A

Note that full information on the approval of the study protocol must also be provided in the manuscript.

Field-specific reporting

Please select the one below that is the best fit for your research. If you are not sure, read the appropriate sections before making your selection.

☒ Life sciences ☐ Behavioural & social sciences ☐ Ecological, evolutionary & environmental sciences

For a reference copy of the document with all sections, see nature.com/documents/nr-reporting-summary-flat.pdf

Life sciences study design

All studies must disclose on these points even when the disclosure is negative.

Sample size	Sample sizes were often determined under the supposition that a biologically significant difference in the parameters evaluated would be 30%, with a sigma estimate of 10–20% of the predicted mean. The conventional values of.05 and 0.8 were assigned to Alpha and Beta, respectively. When appropriate, one-way ANOVA or the Student's t-test are used in the statistical analyses that are presented.
Data exclusions	No data were excluded
Replication	Each experimental test/study is supported by replicate analysis (2-3 times)(except the monkey experiments). All attempts at replication were successful.<br>The experiment on the anti-VEGF protein level and the co-immunofluorescent analysis of coronal sections of the NHP retina involved only one monkey. However, twelve monkeys were utilized to assess the treatment efficacy for nAMD. The efficacy results have consistently replicated and confirmed the anti-VEGF protein level and the co-immunofluorescent results.
Randomization	This study uses mice, rabbits and monkeys as experimental animals. For each cohort within the various experiments, inbred mice were randomly selected from a group of mice of the same age and sex plus similar weight to standardize the control and experimental groups as much as possible. The rabbit and monkey experiments were conducted using a randomised design, with all subjects being of similar weight and age. For the monkey efficacy experiments, the number of male and female participants was split equally among the groups.
Blinding	Data collections were blinded for the initial steps of the study. Animal studies were carried out by multiple investigators to mitigate bias: S.Luo and Y.F.Qing performed mouse injections, who were blinding to the treatment groups by coding; S.Luo, H.Jiang, Q.W.Li and Y.F.Qing performed rabbits and monkeys injections; S.Luo and H.Jiang prepared the tissues and executed the immunofluorescence workflows; and

X.Ke, Q.Zheng and X.Sun performed data analysis and interpretations . In addition, all study parameters are based on quantitative data and therefore did not require blinding to rule out investigator bias.

Reporting for specific materials, systems and methods

We require information from authors about some types of materials, experimental systems and methods used in many studies. Here, indicate whether each material, system or method listed is relevant to your study. If you are not sure if a list item applies to your research, read the appropriate section before selecting a response.

Materials & experimental systems

n/a	Involved in the study
☐	☒ Antibodies
☐	☒ Eukaryotic cell lines
☒	☐ Palaeontology and archaeology
☐	☒ Animals and other organisms
☒	☐ Clinical data
☒	☐ Dual use research of concern
☒	☐ Plants

Methods

n/a	Involved in the study
☒	☐ ChIP-seq
☒	☐ Flow cytometry
☒	☐ MRI-based neuroimaging

Antibodies

Antibodies used

1) anti-eGFP, 1:800, Invitrogen, #A11122;
2) anti-Rhodopsin, 1:200, Invitrogen, #PA5-85608;
3) anti-Arcchis hypogaea, 1:100, Invitrogen, #L32460;
4) anti-Calbindin D28K, 1:100, Invitrogen, #PA1-931;
5) anti-GFAP, 1:200, Invitrogen, #MA5-12023;
6) anti-CHX10, 1:100, Invitrogen, #PA5-85404;
7) anti-RPE65, 1:200, Invitrogen, #PA5-110315;
8) anti-RBPMS, 1:200, Invitrogen, #PA5-31231;
9) anti-PROX1, 1:200, Invitrogen, #PA5-85552;
10) anti-GS, 1:200, Invitrogen, #MA5-27749;
11) anti-VEGFR1 antibody, R&D, #MAB321;
12) HSPG2-specific polyclonal antibody, Proteintech, #1967575-01;
13) goat anti-rabbit antibody, 1:5000, Beyotime, #A0208

Validation

All commercially available antibodies are commonly used and were validated by the suppliers, as documented below:
1) https://www.thermofisher.cn/cn/zh/antibody/product/GFP-Antibody-Polyclonal/A-11122;
2) https://www.thermofisher.cn/cn/zh/antibody/product/Rhodopsin-Antibody-Polyclonal/PA5-85608
3) https://www.thermofisher.cn/order/catalog/product/cn/en/L32460
4) https://www.thermofisher.cn/cn/en/antibody/product/Calbindin-D28K-Antibody-Polyclonal/PA1-931
5) https://www.thermofisher.cn/cn/en/antibody/product/GFAP-Antibody-clone-ASTRO6-Monoclonal/MA5-12023
6) https://www.thermofisher.cn/cn/en/antibody/product/CHX10-Antibody-Polyclonal/PA5-85404
7) https://www.thermofisher.cn/cn/en/antibody/product/RPE65-Antibody-Polyclonal/PA5-110315
8) https://www.thermofisher.cn/cn/en/antibody/product/RBPMS-Antibody-Polyclonal/PA5-31231
9) https://www.thermofisher.cn/cn/en/antibody/product/PROX1-Antibody-Polyclonal/PA5-85552
10) https://www.thermofisher.cn/cn/en/antibody/product/Glutamine-Synthetase-Antibody-clone-GT1055-Monoclonal/MA5-27749
11) https://www.rndsystems.com/cn/products/human-vegfr1-flt-1-antibody-49560_mab321
12) https://www.ptgcn.com/Products/HSPG2-Specific-Antibody-19675-1-AP.htm
13) https://www.beyotime.com/product/A0208.htm

Eukaryotic cell lines

Policy information about cell lines and Sex and Gender in Research

Cell line source(s)

HEK293T cells (ATCC, #CRL-3216); ARPE19 cells (ATCC, #ATCC®CRL2302TM); HeLaRC32 cells (ATCC, #CRL-2972TM) ;

Authentication

HEK293T :https://www.atcc.org/products/crl-3216
ARPE19 cells: https://www.atcc.org/products/crl-2302
HeLaRC32 cells: https://www.atcc.org/products/crl-2972
No further authentication was performed.

Mycoplasma contamination	Cell lines were negative for myoplasma
Commonly misidentified lines (See ICLAC register)	No commonly misidentified cell lines were used in the study.

Animals and other research organisms

Policy information about studies involving animals; ARRIVE guidelines recommended for reporting animal research, and Sex and Gender in Research

Laboratory animals	Six- to eight-week-old male C57BL/6J mice, twelve- to thirteen-week-old male New Zealand White rabbits with weight ranging from 2.0 to 2.5 kg (Dashuo Laboratory Animal Technology Co., Ltd., China), and two-year-old Cynomolgus monkeys with weight ranging from 2.5 to 5.0 kg (West China-Frontier PharmaTech Co., Ltd., Tianfu Drug Research Center, SYXK(Chuan)2021-238) were used in this study. The mice and rabbits were housed under specific pathogen-free (SPF) conditions (70~74F with humidity at 35~45%) at West China school of Pharmacy, Sichuan University, with a 12-hour light/12-hour darkness cycle. Mice and rabbits were fed normal chow (#1010088, #1010070). Cynomolgus monkeys were housed in West China-Frontier PharmaTech Co., Ltd., Tianfu Drug Research Center.
Wild animals	No wild animals were used in the study.
Reporting on sex	Sex was not considered in the AAV efficacy study, other than for balancing the study and control groups (rabbits and monkeys). Both sexes were used. For mice study, only male mice were used, following a published protocol.
Field-collected samples	No field collected samples were used in the study.
Ethics oversight	All animal (mice and rabbits) procedures described in this study were approved by the Committee on the Ethics of Animal Experiments of Sichuan University . The cynomolgus monkeys were housed in West China-Frontier PharmaTech Co., Ltd., Tianfu Drug Research Center. The institution is accredited by AAALAC International (Association for Assessment and Accreditation of Laboratory Animal Care International). The experiments conducted with cynomolgus monkeys followed the regulations of the Institutional Animal Care and Use Committee (IACUC) and the Guide for the Care and Use of Laboratory Animals (8th Edition). The animal experiments were designed in accordance with the appropriate guidelines. This experiment has been approved by the Institutional IACUC under the approval number: IACUC-SW-A2021046-P004-01.

Note that full information on the approval of the study protocol must also be provided in the manuscript.

Plants

Seed stocks	N/A
Novel plant genotypes	N/A
Authentication	N/A
